# Notes of an European Tour

**Published:** 1846-01-01

**Authors:** F. H. Hamilton

**Affiliations:** Professor of Surgery in Geneva Medical College


### BUFFALO MEDICAL JOURNAL

Vol. 1. JANUARY 1, 1846. No. 8.

### Notes of an European Tour.---No. 8.

Communicated for the Buffalo Medical Journal, by F. H. HAMILTON, M. D., Professor of Surgery in Geneva Medical College.

ITALY.

I saw also in the "Ospedale Maggiore" a number of cases of the "Lepra Tuberculosa," the Tsarath of the scriptures, or the Greek Elephantiasis, differing wholly from the Arabian Elephantiasis, or any other form of disease I had ever seen. I supposed this disease peculiar to tropical climates, and was surprised to find so many cases in a latitude so far north. I did not see any suffering from the last and most horrible stage, the stage of ulceration, sloughing, &c., but there were specimens sufficiently hideous; from a few brownish knobs scattered upon different parts of the face, to a total disfigurement and obliteration of all the features.—The patients were generally able to sit up and converse, yet their spirits were exceedingly depressed. Like Pellagra it is generally incurable.

In the surgical wards I found several cases of fractures, and generally the straight splints were employed: the dressings, simple and light. A case of fractured clavicle, I was pleased to find secured by a sling, axillary pad, and a single roller; a practice, the superiority of which to the complicated bandages of Desault, is now very generally admitted; indeed, I do not recollect to have seen Desault's apparatus applied since I have left home. Another fact which I now recollect to mention, is, that here, as elsewhere in Europe, where the straight splints are employed for extension and counter-extension, very moderate force is applied, much less than I have been accustomed to see used at home; and I have never seen those ulcerations produced by the extending means, which an opposite plan is so liable to produce. Continental Surgeons seem to understand that nothing is gained by violent extension but ulceration! Through Dr. Capelli, I enquired of several patients if the extension was painful, and they uniformly replied that it was not.

The Alms House of Trivulzi is a private charity, founded in 1771, by Prince Antonio Trivulzio, who not only made a liberal endowment, but gave up his own palace for the hospice; since which time it has received additional endowments, and the building has been much enlarged. It is a strange feature in the religious character of Catholic Europe, that while both noble and king combine to oppress and rob the poor, they are constantly found endowing public charities of all kinds with a princely munificence, of which we can scarcely conceive. The hospitals and hospices in these countries, are every where upon a most magnificent scale, and their income often immense—and yet the streets, and highways, and churches are forever thronged with miserable beggars. None are admitted at Trivulzi but those who are over 70 years of age. The number is now nearly 600.

Having procured a "claesse," and a cicerone, we drove through the fine "corso di porta orientale," to get a view of the ancient lazaretto just without the eastern gate. It is an immense building, enclosing a square court, being one mile in circumference. We could not get admission, which I regretted the more as its history is associated with that of the plague, and its vast and crowded cloisters have witnessed some of the most thrilling scenes of human misery. It is now unoccupied.

From the Lazaretto we drove to the Duomo, next to St. Peters Church in point of size, and the most elegant and impressive gothic Cathedral in the world. I have already disclaimed all intention to edify you with architectural descriptions, and in pursuance of my determination shall only add that it is built of the finest white marble, is covered with a forest of richly wrought pinnacles upon which already stand 3000 statutes, and 1500 are yet to be carved and erected. Ever since the first stone was laid, in the year 1386, some portion of the scaffolding has been standing, "and the sound of the mallet and the chisel have never ceased."

Within, all is rich and solemn, grand and impressive. Beneath the church is the "scusolo" containing the sepulchral chapel of the patron saint Borromeo, whose body may be seen enclosed in a gorgeous golden sarcophagus inlaid with silver; he is clad in his full pontificals, and his dry and mouldering face gives a ghastly look from underneath his gilded mitre. Around the shrine are suspended a great variety of gold and silver jewels, which are the votive offerings brought to the saint by those whom his intercession has cured of diseases. I thought that while the dead saint was reaping a golden harvest for the benefit of the living priests, the physician, to whose skill alone the recovery was due, probably never received, perhaps never asked, a farthing—more of this anon.

Returning to the upper church, the guide pointed to a reliquary hanging above the altar, "which contains," says he, crossing himself devoutly, "one of the nails of our Saviour's cross!"

From the Cathedral we drove through many of the principal streets, called "corsos," some of which seemed a continued succession of palaces, and might well entitle Milan to its ancient epithet "la grande;" and having given a passing inspection to Napoleon's triumphal arch, the ampitheatre, the celebrated opera house La Scala, the latter of which I saw again in the morning most brilliantly illuminated, I returned to my Hotel to discuss an excellent dinner, and ruminate upon all I had seen in the now capital of the Austro-Italian dominions—under Napoleon, the capital of the Kingdom of Italy, and the place which, in the days of Knighthood, clad in steel panoply half the knights of Christendom. "Well was he armed from head to heel, in mail and plate of Milan steel."

Leaving Milan from the south west, we came along the northern bank of the great "naviglio" or canal, which reaches from Milan to the river Ticino at Pavia, a distance of 20 miles, and which commenced many centuries since, was completed by Napoleon during his occupation of

Italy. On each side the country stretches out in a wide and uninterrupted level, intersected only by occasional ditches, made to drain off the water, which, whenever the rains are considerable, settles in a broad lake over a great portion of the meadows. The soil, although humid, is exceedingly fertile, yielding often two or three crops of hay in the year; but rice and corn are the principal products. As might be expected from a soil so moist and fertile, miasmatic fevers abound, with a considerable proportion of pellagra, dropsies, &c., and the peasantry whom I saw along the road, presented generally an unhealthy appearance—they were *always* wretchedly clad!

The ancient fortress of Binaseo is near the road, half way to Pavia; it is remembered as the castle of the cruel and superstitious Duke Filippo Maria, who, instigated by an unjust jealousy and certain astrological signs, caused his wife, the beautiful Beatrice, to be placed upon the rack, and afterwards beheaded on the night of the 13th of September, 1418. A few miles beyond, we were pointed to the plain upon which Francis the First, in 1525, was, after a most fierce and bloody battle, made prisoner by Charles the Fifth. Terminating an avenue, some distance from the road, rises the vast and gorgeous Monastery erected by the pious zeal of the murderous Galeazzo, who hoped to expiate his terrible crimes by building a temple wherein to stall and fatten monks. It was from an upper room in this monastery that Francis the First, then a prisoner, wrote to his mother, "all is lost save honor."

Pavia! Pavia! suddenly exclaimed my only companion, a Genoese Merchant, as we drove under a heavy stone gateway, and began to thunder through streets dark, and scarcely wider than the Vettura, turning again and again as if threading a labarynth. It was a cold, raw, rainy day, the rain, a Lombardy rain, and the wind, a Tramontane! and we had shut ourselves up closely within the Vettura and crept snugly within our cloaks; and dreaming only of a warm dinner and a blazing fire, I paid no farther attention to my friend's announcement than to nod my head in assent. At length after a final and unusually noisy cracking and flourishing of the whip, by both postilion and vetturino, we drew up to the "Grande Albergo della Lombardia." But I have learned never again to put trust in a name, for a more filthy, cold, and comfortless looking place never vexed a shivering traveller. Italian Inns, with their large airy rooms and stone floors, and light faggot fires, are sad trials in such weather for the classic romance, as well as tempers of foreigners. Let no one who journeys for either pleasure or health, travel in Italy, especially the north of Italy, in winter or spring. I would rather endure a whole winter of Canadian storms, than one week of a wet tramontane, relaxed and enfeebled as the body often is by the preceding days of hot sun and sultry southern winds, and accompanied, as they always are to the traveller, with an entire absence of even the *necessary* in-door comforts.

We had had only two hours by agreement to remain, one of which I was compelled to lose in fruitless attempts to get warm, and in swallowing a mawkish soup, a piece of boiled hen, bad wine and stracheno cheese; a piece of Sardinian ham only was excellent. For two things the petty tyrant of Sardinia shall be praised—for his fine breed of hogs, and his anchovies! If I love him for any thing else I will tell you.

One hour only was therefore left me to see Pavia—Pavia, the city of 500 towers—the ancient Capitol of the Lombard Kings—the second imperial city of the Lombardo Venetian States—one hour to see its great University where taught Spallanzini, who frightened the poor superstitious peasantry of Lombardy almost into sending him to the inquisition—where Fontana lectured, and Volta *electrized* his auditors—where Frank and Scarpa lived and labored. Here was work enough for one hour! Sheltered with my india rubber coat, cap and umbrella, I sallied out with a commissionaire, exciting every where as I passed, as much curiosity as if one of the ancient leathern clad Lombards had appeared among them; the boys ran after me, the men stopped and looked, and the women laughed—*en passant*, the black silk veil which the Pavian women wear almost universally upon their heads, an ancient costume, gives them all a peculiar, widowed appearance, not altogether uninteresting.

The University buildings are extensive, and I could only *run* through them, stopping a moment to read upon a couple of marble monuments in one of the courts, the names of Fontana and Spallanzini. The anatomical museum is smaller than I had expected to see. One vascular preparation of the arm was shown me, in excellent preservation, prepared by Spallanzini himself. It could not be bought, nor indeed was there any thing with which, for money, the custode would part. Authorized to make anatomical purchases for Geneva, wherever those having value can be obtained, I have looked every where among private and public museums most zealously, and have been surprised thus far to find none which can be purchased. Wherever good preparations are found they are valued, and being generally the property of well endowed establishments, they are not compelled to, and will not part with them.

The number of students now attending I have forgotten—it is not large: the term of study is five years.

The hospital where Scarpa labored I did not see, but I learn that it is small and uninteresting.

In my rambles I passed the ruins of the castle in which Petrarch once lived, and caught a few glimpses of some of those very tall and curious looking stone towers, which were first used for purposes of defence; their number, once said to be 525, is now reduced to four or five.

The river Ticino washes the eastern wall of Pavia, soon after crossing which we were upon the banks of the classic Po, a broad and majestic river, winding between low and marshy banks, which are often flooded to many miles in each direction. The bridge is called a "pont volant"—flying bridge—being composed of flat boats fastened to each other, side and side.

A few miles beyond we drove through Casteggio, whose fortress was considered in ancient warfare impregnable, but was delivered over to Hannibal by the traitor Dasius. Under its walls also was fought in 1800 the famous battle called the battle of Montebello, in which Napoleon gained a most signal victory.

At Voghera we entered Sardinia, ten miles beyond which we passed through Tortona, one of the most ancient cities of northern Italy. To the right of Tortona lies the plains of Marengo! a bloody and memorable field.

Sleeping at Novi, we commenced at 6 o'clock the ascent of the Appenines. The road is smooth and the rise gradual, the horses often trotting a mile at a time. At midday we had crossed the summit and were beginning our descent upon the southern slope, with the Mediterranean and "Genoa the superbe" in full view.

